# Cellular code for mnemonic pattern separation in the human hippocampus is revealed by false memories

**DOI:** 10.64898/2026.09.18.752786

**Published:** 2026-09-20

**Authors:** Natalia Kurilenko, Clayton Mosher, Sophia Cheng, Yousef Salimpour, Jonathan Daume, Chrystal M. Reed, William Stanley Anderson, Taufik A Valiante, Adam N. Mamelak, Ueli Rutishauser

## Abstract

Theoretical models propose that pattern separation, a computation thought to be implemented by the hippocampus, allows us to differentiate between familiar and novel items. However, whether pattern separation operates in the human hippocampus remains contested, with no established single-cell correlate. We recorded the single neuron activity of 3506 neurons in the human brain while 97 patients performed a recognition memory task in which novel images similar to previously seen images led to false memories and associated behavioral errors. We identified two kinds of memory selective neurons distributed across the brain: those responding differently to falsely recognized novel and correctly recognized familiar images in a manner compatible with pattern separation, and the other signaling the subject's choice. At the population level, these cells predicted mnemonic ground truth in the hippocampus and the decision in the pre-supplementary motor area, illustrating the progression from mnemonic signals to decisions. Removing pattern separation-signaling cells abolished the continuous memory strength gradient present in the hippocampus, suggesting a role of these cells in separating memories of different strength. These results establish a single cell correlate for mnemonic pattern separation in the human hippocampus and show its behavioral relevance in episodic memory.

## Full Text Availability

The license terms selected by the author(s) for this preprint version do not permit archiving in PMC. The full text is available from the preprint server.

